# Multifocal Infarction Along the Alimentary Canal in the Context of Ostensible Salmonellosis: A Case Report

**DOI:** 10.7759/cureus.69707

**Published:** 2024-09-19

**Authors:** Caleb Bryson, Chirag Lodha, Stanley Miller

**Affiliations:** 1 Infectious Disease, Edward Via College of Osteopathic Medicine, Spartanburg, USA; 2 Internal Medicine, Edward Via College of Osteopathic Medicine, Spartanburg, USA

**Keywords:** acute mesenteric ischemia (ami), colonic neuroendocrine tumor, hypercoagulable, salmonella enteritis, shwarzman reaction, venous mesenteric ischemia

## Abstract

Ischemic and/or infarction events of the alimentary canal are uncommon but potentially disastrous injuries of the digestive system that often portend a poor prognosis. Alimentary ischemia occurs when the vascular supply to one of the component conduit organs is disrupted or blocked, resulting in decreased tissue perfusion, subsequent necrosis, perforation, and even death if proper perfusion is not restored. We report a case here of a 67-year-old female who originally presented to the emergency department (ED) with nausea, vomiting, diarrhea, and progressively worsening abdominal pain. Conservative therapies that were initially employed failed to provide lasting symptom relief, and the patient was admitted for a more in-depth diagnostic workup and closer monitoring. During subsequent days of her resulting hospital stay, the patient had a positive result for *Salmonella* spp. on a stool PCR assay, an increasing leukocytosis, and the presence of several other worrisome laboratory abnormalities. Despite appropriate antibiotics and aggressive fluid resuscitation efforts, the patient’s abdominal pain and laboratory profile continued to progressively worsen. At one point, the patient’s condition perilously worsened, necessitating an emergent exploratory laparotomy. During the course of this surgery and subsequent surgeries, the patient was found to have multiple areas of infarction present including at her esophagus, stomach, duodenum, proximal jejunum, and right colon. Additionally, evidence of a metastatic neuroendocrine tumor of gastrointestinal (GI) origin was also incidentally found. Several subsequent surgical operations were required to repair the extensive tissue damage that the patient had sustained, and the patient’s resulting hospital stay was complicated repeatedly by several different secondary infections and surgical complications. Attempts to determine the underlying cause for the ischemic events this patient experienced failed to yield definitive results, and no evidence for any arterial insufficiency or emboli was ever discovered. Despite this, a review of the histopathologic and laboratory findings from the tissue resected from the patient did find information to suggest that a relatively localized but severe venous thrombotic process likely occurred in the patient’s alimentary vasculature that directly led to her presentation. Venous thrombosis of the mesenteric vessels and in the other vascular planes of the alimentary canal is often insidious in its presentation and poses a unique diagnostic challenge to clinicians. This case is significant because it illustrates the diagnostic complexity and difficulty imposed by mesenteric ischemia, especially cases resulting from mesenteric venous thrombosis (MVT) due to their often more indolent and atypical presentation. In short, a high level of clinical suspicion and familiarity with this ailment and its risk factors should be maintained because, in the absence of timely intervention, significant morbidity and/or mortality are likely to result.

## Introduction

Acute mesenteric ischemia (AMI) is traditionally defined as a medical condition that occurs whenever perfusion to the intestines by the mesenteric vasculature is suddenly impaired; this vascular compromise will result in progressive intestinal ischemia and subsequent infarction if adequate perfusion to the affected area is not restored [1]. Due to this, AMI is a life-threatening intestinal emergency that often portends a poor prognosis and a high mortality rate ranging between 50% and 100% in advanced cases [2]. Furthermore, in the past, AMI was regarded as a relatively rare diagnosis, accounting for less than 1% of patients presenting with an “acute abdomen” [2]. However, in recent years the incidence of AMI is believed to have increased and now accounts for up to 10% of cases of “acute abdomen” in patients greater than 70 years old; with America’s patient population growing older and with the incidence of AMI on the rise in these elderly populations, the prompt diagnosis and management of AMI should be increasingly prioritized by clinicians [2].

AMI can result from any condition that can cause an acute decrease in blood flow through the mesenteric vasculature; mesenteric arterial embolisms are believed to account for the majority of cases (50%), followed by mesenteric arterial thrombosis (15%-25%), and mesenteric venous thrombosis (MVT) being the least common cause of the condition (5%-15%) [2]. An additional form, nonocclusive mesenteric ischemia (NOMI), has also been observed and is believed to be the result of splanchnic vasoconstriction, often in the context of heart failure or marked hypotension secondary to hypovolemia or other causes of shock; epidemiologic data documenting the occurrence rates of NOMI are sparse [2]. The superior mesenteric artery (SMA) is the most commonly affected vessel in cases of AMI and is especially prone to AMI due to emboli because of its large diameter and narrow takeoff angle as it branches off the abdominal aorta [2]. Importantly, risk factors for AMI are comorbidities that are also increasingly prevalent in the American patient population, with conditions like coronary artery disease, peripheral artery disease, atrial fibrillation, arterial hypertension, congestive heart failure, and inherited and acquired hypercoagulable states as notable examples [1].

## Case presentation

A 67-year-old female presented to the emergency department (ED) with a chief complaint of persistent nausea, vomiting, and diarrhea that had been present for four days. The patient had been seen twice in the ED on preceding days for these same symptoms; on both of those occasions, a thorough workup showed that the patient had a mildly elevated lactic acid level and slight leukocytosis (Table 1), but no definitive cause was found for the patient’s symptoms; additionally, the patient did report that one month previously, she had been admitted for “enteritis” that she recovered from after being given intravenous (IV) fluids. The patient’s past medical history was significant for previously diagnosed type 2 diabetes, hypertension, and hyperlipidemia, but none of these conditions were believed to be significantly involved with her current presentation and gastrointestinal (GI) distress. After close monitoring in the ED, and once apparent pathologies were ruled out, the patient was discharged home after showing improvement on IV fluids and antiemetics. 

The following day, the patient returned to the ED complaining of the same symptoms, as well as new-onset right lower quadrant abdominal pain. After this presentation, the patient was admitted to an in-patient unit for further workup and evaluation because the patient was found to be febrile and because her symptoms had progressed in severity to the point where IV morphine and IV ondansetron were required. During this admission, the patient had a noncontrast CT scan of her abdomen and pelvis; due to a history of an anaphylactic allergic reaction to iodinated CT contrast dyes, no contrast was used in any of her imaging. The scans that were performed found no apparent abdominopelvic pathology, with the exception of a non-obstructing left nephrolithiasis, and the patient was treated with empiric metronidazole and ciprofloxacin under a presumptive diagnosis of enteritis and was given further IV fluids. A stool sample was also taken for polymerase chain reaction (PCR) analysis to evaluate for an underlying infectious etiology for the patient’s symptoms and was found to be positive for *Salmonella *and negative for *Clostridium difficile*. A culture of the *Salmonella*-positive stool was sent for culture but never resulted in any growth; for this reason, no subspecies or serovar testing to further evaluate the type of *Salmonella *found in the patient was performed. Metronidazole was discontinued after the patient’s stool was found to be positive for *Salmonella*, but the IV ciprofloxacin the patient had been receiving was continued. The patient’s fever and leukocytosis had resolved by the 4th day of her admission, and she was discharged that day on oral ciprofloxacin and instructed to continue her antibiotics until she had completed a total of seven days of antibiotic therapy for this ostensible case of Salmonella gastroenteritis.

**Table 1:** Patient labs WBC: White blood cell; HgB: hemoglobin; PLT: platelets; CRP: C-reactive protein; PT: prothrombin time; INR: international normalized ratio

Serum lab	Patient value on 10/30/2022	Patient value on 10/31/2022	Patient value on 11/04/2022	Patient value on 11/07/2022 (morning)	Patient value on 11/07/2022 (afternoon)	Reference value	Units
Creatinine	1.35	1.44	1.33	1.33	2.29 (H)	0.57-1.00	mg/dl
Calcium	11.7	N/A	N/A	N/A	7.4	8.9-10	mmol/L
WBC	19.1 (H)	24.6 (H)	17 (H)	25.6 (H)	18.2 (H)	3.7-10.6	K/uL
HgB	14.4	N/A	15.3	9.7 (L)	12.3 (L)	13-18	g/dL
PLT	439	374	473	300	164	140-450	K/uL
Lactic acid	2.4 (H)	1.8	1.7	4.0 (H)	>10.0 (H)	0.3-2.1	mmol/L
CRP	-	-	7.2 (H)	15 (H)	-	0.0-0.6	Mg/dl
D – Dimer	-	-	5.39 (H)	-	-	0.0-0.45	Ug/mL FEU
PT	-	-	-	20.5 (H)	-	12.7-15.1	seconds
INR	--	-	-	1.8 (L)		2.0-03.0	

The patient re-presented to the ED the following day with a relapse and progression in the severity of her symptoms, despite reporting she had been compliant with taking her antibiotic as prescribed. At this presentation, the patient’s nausea, vomiting, and diarrhea had resumed, and her abdominal pain had progressed to where it was now diffuse but most intense in her upper abdomen and epigastric area. On exam, the patient was noted to appear visibly ill and with a mildly distended abdomen; furthermore, mild tenderness to palpation was also found in the patient’s upper abdomen. The patient was also found to be hypoxic and tachycardic in the ED, but testing for mycoplasma, respiratory syncytial virus (RSV), influenza, and COVID-19 were all negative. At this time, the patient was also found to have elevated prothrombin time (PT) and international normalized ratio (INR) values, as well as an elevated D-dimer (Tables 1-2). Due to her hypoxia and these abnormal lab values, a ventilation-perfusion (VQ) scan was then ordered to rule out a pulmonary embolism (PE); this VQ scan discovered the patient did have two small perfusion defects in her lungs, but these were judged by the radiology department to be at low risk for having been caused by a PE. Again, no contrast-aided CT angiography was utilized due to the patient’s intolerance to CT contrast dyes. A repeat noncontrast CT scan of the patient’s abdomen and pelvis was then performed that showed the patient had signs of duodenitis; following this, the patient was again placed on IV ciprofloxacin and metronidazole and admitted for further evaluation and treatment.

**Table 2:** Patient coagulation labs PT: Prothrombin time; INR: international normalized ratio

Coagulation labs	Patient value	Normal range
D-Dimer	5.39	0.0-0.67 Ug/mL FEU
PT	20.5	12.7-15.1 seconds
INR	1.8	2.0-3.0

Over the following three days, the patient remained afebrile but had signs of a persistent leukocytosis and a rising C-reactive protein (CRP) level (Table 1); due to this, her antibiotic regimen was changed with IV ciprofloxacin being replaced by IV ceftriaxone in combination with continued IV metronidazole. Several sets of blood cultures were obtained over these days, but none ever yielded a positive result. On the 4th night of the patient’s second hospitalization, the patient’s hemoglobin dropped acutely from 12 to 9.7, and the patient also became hypotensive; however, the patient’s blood pressure normalized once IV fluids were administered. An infectious disease consult was placed later that day due to the patient’s lack of clinical improvement and the patient’s antibiotic coverage was further changed to IV cefepime and IV metronidazole. The next morning, the patient suffered what appeared to be an aspiration episode that necessitated a rapid response; during this time, the patient was noted to have black stool, increasing abdominal distension, and dark emesis was suctioned as the patient was intubated. The patient was successfully resuscitated, and another CT scan of the patient’s abdomen and pelvis was performed due to concerns for GI bleeding. The CT scan revealed the presence of persistent thickening and inflammation around the patient’s duodenum, and free fluid was visible in the patient’s right upper quadrant, suggesting a perforated duodenal ulcer. General surgery was consulted, and an exploratory laparotomy was then performed that found large portions of the patient’s duodenum and proximal jejunum were necrotic. A large duodenal perforation with surrounding bile peritonitis and an ischemic right colon were also found, necessitating an emergent right hemicolectomy. The patient was then transferred to a tertiary care center and underwent a Whipple procedure (gastroduodenopancreatectomy) to remove the remaining necrotic areas found throughout her GI tract. Subsequent histologic analysis performed on the tissues resected during this surgery found multiple areas of ischemia and infarction consistent with those seen in cases of AMI; furthermore, a previously undiagnosed metastatic neuroendocrine tumor was also discovered in two of the 14 resected peripancreatic lymph nodes. Cytology performed on the tumor cells showed they were grades 1-2, indicating they were well-differentiated (low grade) to intermediately differentiated (intermediate grade).

The patient survived her extensive operations, and her postoperative hospitalization was complicated twice by two separate instances where intrabdominal fluid collections that had formed became infected. The first was tapped and grew* Candida albicans*, prompting the patient to be placed on micafungin. Around two weeks later, a second intraabdominal fluid collection was spotted on a follow-up CT; this collection was also tapped and grew methicillin-sensitive *Staphylococcus aureus *(MSSA), for which the patient was placed on cefazolin. In the process of determining the source of those intraabdominal fluid collections, an esophagogastroduodenoscopy (EGD) was performed to see if there was a persistent leak or fistula that had formed along the patient’s GI tract. No perforation or fistula was visualized on the EGD, but areas of ulceration, possibly due to necrosis, were noted in the patient’s distal esophagus and proximal jejunum, evidenced by the patient's rising lactate (Table 1). Despite these complications, the patient recovered with appropriate treatment, and after several months in the hospital, she was eventually discharged to a long-term acute care facility to continue her recovery.

## Discussion

This clinical case illustrates how AMI, especially cases caused by MVT, can insidiously present with nonspecific symptoms that steadily worsen over time, eventually resulting in an acute decompensation in a patient's condition and significant morbidity and/or mortality. There are several factors that support that the patient described here experienced AMI due to MVT; one specific factor is how the patient’s symptoms manifested over several days and progressively worsened but were intermittently reduced by the provision of IV fluids and antiemetic therapies in the ED. AMI due to an arterial occlusion would have presented with an acute onset of severe, unremitting abdominal pain that was unresponsive to fluid resuscitation [2]. Other evidence supporting that this patient’s condition was due to MVT was noticeable elevations in the patient’s D-dimer, PT, and INR values. These labs were observed in the patient during her hospitalization soon before she went unresponsive, was observed having black vomit and black stool, and had to be intubated; together these lab abnormalities and the patient’s clinical presentation around the same time support the idea that some degree of the patient’s symptoms occurred as a result of an underlying coagulopathy.

Common models explaining the pathogenesis of hypercoagulable states and associated thrombosis and/or embolus center around the idea of “Virchow’s triad;” the three components of this triad are as follows: alterations in blood flow (i.e., stasis), vascular endothelial injury, and a hypercoagulable state (either inherited or acquired) [3]. Alterations in blood flow can often be seen in patients who are confined to bed rest and unable to ambulate, as well as in patients with atherosclerotic vessel narrowing; the altered circulatory pattern caused by these conditions and conditions like them can result in a loss of normal venous return from the extremities, leading to venous stasis [3]. Furthermore, in the case of atherosclerotic disease, the altered circulatory patterns that occur can cause increased turbulence that can then result in endothelial damage, especially at vascular branching points like the carotid and iliac bifurcations [3]. A second component of Virchow’s triad is endothelial injury; this condition can have numerous etiologies, but vascular damage due to chronic hypercholesterolemia and prolonged systemic inflammation are among the most common causes of endothelial damage [3]. Damage to vascular endothelium can expose underlying collagen that can then result in platelet activation and clot formation, as well as result in greater endothelial expression of tissue factor (TF), a cofactor of circulating clotting factor VII [3].

A hypercoagulable state, part of Virchow's triad, can result due to congenital mutations in blood components like clotting factors or occur as a result of another disease process or environmental exposure [3]. Examples of inherited hypercoagulability are seen in patients with the Factor V Leiden mutation, prothrombin G20210A mutations, antithrombin III deficiency, and protein C deficiency [4]. Hypercoagulability can also be acquired secondary to other disease processes like disseminated cancer or prolonged systemic inflammation, as well as result from environmental exposures to things like tobacco smoke or certain drugs like estrogen-containing contraceptives [4]. Cancers are well known for increasing the risk of thromboembolism, with around 10%-15% of all patients diagnosed with cancer experiencing some form of venous thromboembolism (VTE); furthermore, current research suggests that patients with pancreatic, lung, gastric, breast, and colon cancer experience the highest incidence of cancer-associated VTE [5]. Current explanations for this increased risk of VTE in patients with a malignant neoplastic process is that cancer cells synthesize and release procoagulants in the nearby tumor microenvironment; the main procoagulants implicated in cancer-associated VTE are cancer procoagulant (CP) and TF [5]. Excessive expression of TF, occurring as a result of endothelial damage, systemic inflammation, or due to cases of malignancy, is an important contributor in the acquisition of a hypercoagulable state and in the pathogenesis of subsequent VTE. TF exerts its effects by binding with circulating clotting factor VII (FVII) and forming an enzymatic complex that initiates the extrinsic pathway of the coagulation cascade by proteolytically activating factor IX and factor X [5]. The initiation of the extrinsic pathway, as described here, leads to systemic hypercoagulability and has been implicated as a major contributor to the pathogenesis of deep vein thromboses (DVT), PEs, and cases of disseminated intravascular coagulation (DIC) [5].

In addition to directly inducing a hypercoagulable state through the release of procoagulants, as previously mentioned, malignancy-induced immunosuppression has been shown to be a major risk factor for multiple infections, including disseminated Salmonella infections and their associated complications [6]. *Salmonella *species are gram-negative enteric bacilli and are traditionally associated in the United States with mild to moderate self-limiting cases of enteritis. Like other gram-negative bacteria, the cell membrane of *Salmonella *bacteria contains lipopolysaccharide (LPS), a multipart outer membrane component that provides integrity to the bacteria and aids bacterial interaction with surfaces; furthermore, LPS is an important bacterial virulence factor that is associated with inducing an intense proinflammatory immune response [7]. Antibiotic therapies targeting gram-negative bacteria can result in large-scale bacteriolysis and a massive subsequent release of LPS into the host’s bloodstream; this influx of LPS into the bloodstream is thought to sensitize tissues to future cytokine stimulation as well as directly initiate immune dysregulation, leading to tissue damage [8]. Current research on the pathophysiology behind the effects of LPS states that LPS likely activates innate immune cells by way of binding to Toll-linked receptors (TLRs); these activated immune cells then release the proinflammatory cytokine TNF-α; TNF-α then stimulates matrix degrading metalloprotease enzymes that then go on to degrade the glycosaminoglycan (GAGs) components of the extracellular matrix of blood vessels [8]. LPS is also believed to sensitize tissue to cytokine stimulation, resulting in a more intense immune response and greater recruitment of immune cells [9]. The net effects of LPS via this increased tissue sensitivity to cytokine stimulation, in combination with the direct tissue damage mediated by TNF-α that it induces, have been described in a process known as the “Shwarzman reaction” and results in leukocytic consolidations around blood vessels, intravascular thrombosis, and the necrosis of venules [8]. This vascular damage results in increased synthesis of TF that can then activate the extrinsic pathway of the coagulation cascade in the same way CPs do as previously described, leading to a hypercoagulable state in some cases [5].

Regardless of the underlying cause, AMI results in damage and subsequent morbidity and mortality by causing an interruption and/or cessation of normal tissue perfusion in affected areas. Tissue ischemia then results and, if allowed to progress without the restoration of normal perfusion, then irreversible damage will be incurred by the mucosal linings in affected areas within six hours [2]. The compromised mucosa then releases inflammatory mediators, prompting infiltration by reactive leukocytes that then release reactive oxygen species (ROS) that further damage the areas of ischemic mucosa. Due to this, the barrier function of the ischemic mucosa is significantly diminished, allowing for bacteria previously sequestered in the alimentary canal to translocate into the peritoneal cavity, leading to gangrene of the intestinal wall and peritonitis [2]. At this point, the resulting peritonitis may result in an ileus, sepsis, multiorgan dysfunction/failure, and death even if appropriate treatment is provided; this is illustrated by research showing that cases of AMI where treatment is delayed have a drastically worse prognosis with an 80%-100% mortality rate being seen in many cases of AMI where treatment is delayed 24 hours or more after symptom onset [2].

Despite modern medical advancements in diagnostic technologies and surgical techniques, AMI remains a challenging condition to diagnose and treat; a major contributing factor to the mean mortality rate of 50%-100% that is seen in advanced cases of AMI is a diagnostic delay lasting eight hours on average [2]. This delay often results in extensive tissue damage and necrosis in the affected areas. Despite the severity of tissue damage and associated symptoms seen at later stages, AMI often initially presents with nonspecific abdominal symptoms like nausea, vomiting, tenderness to palpation, as well as pain patterns and abdominal distention that may closely resemble more common abdominal pathologies like appendicitis, severe constipation, peptic ulcer disease, or bowel obstructions [10]. However, one classic symptom regularly reported by patients suffering from AMI does stand out as notable: “pain that is out of proportion with the physical exam” [10]. Based on that, AMI should be seriously considered and appropriately ruled out or treated in patients presenting with “pain out of proportion with the physical exam,” especially elderly patients above 70 years old with a history of cardiovascular or hypercoagulable risk factors [10].

There is a degree of variability in the clinical presentation of AMI that is dependent on the underlying cause of the ischemia; specifically, AMI due to venous thrombosis has unique aspects in its clinical and histologic presentation that help distinguish it from AMI due to other causes [11]. In cases of AMI due to arterial occlusion, patients commonly report abdominal pain that is extremely severe, out of proportion with physical exam findings, sudden in onset, and constant [11]. This is in contrast to AMI due to venous thrombosis, where patients often describe their initial symptoms as colicky mid-abdominal pain that progressively worsens and becomes more constant over a variable period of hours to days; due to this, it has been observed that up to 75% of patients experiencing AMI due to venous thrombosis have symptoms that had been present for more than 48 hours before seeking care [11]. In cases of AMI due to arterial occlusion, an acute blockage of the supplying artery is blocked suddenly, resulting in an acute onset of severe symptoms [11]. In contrast, AMI due to venous thrombosis occurs due to a thrombotic process that initially occludes the small venous arcades in the intestine before advancing into increasingly larger venous structures; this thrombosis results in venous congestion and swelling in affected areas that eventually progresses to the point where a massive increase in pressure is exerted by the distended veins, disrupting arterial flow into the area and leading to ischemia and/or infarction [11]. AMI due to MVT also commonly causes a dramatic decrease in venous return from the intestines; this, in turn, creates localized areas of venous stasis and secondary bowel wall edema that can then lead to significant fluid sequestration in the area around the affected intestinal veins, resulting in relative hypovolemia and necessitating fluid resuscitation [10]. Due to these unique aspects of its pathogenesis, AMI due to venous thrombosis has a more protracted onset and progressive nature in its presentation [11]. AMI due to venous thrombosis also has been shown to exhibit a unique pattern of pathologic changes that help distinguish it from AMI due to arterial occlusion; affected tissue areas of bowel infarction due to MVT show patchy discontinuous areas of “dusky cyanosis that gradually fade into adjacent areas of normal tissue”; this is in contrast to cases where bowel infarction is secondary to acute arterial occlusion, where the affected tissues exhibit continuous areas of infarction with a sharply demarcated transition point observed between the affected area and unaffected surrounding tissues [12].

Further evidence supporting that this patient’s symptoms were secondary to MVT is revealed in the pathologic evaluation of GI tissues resected during the exploratory laparotomy this patient underwent. The resected samples showed evidence of multiple focal areas of diffusely dusky red-brown tissue with variable levels of necrosis interspersed throughout several vascular planes, including areas in the stomach, duodenum, pancreas, jejunum, and colon. These histologic changes closely resemble the pattern of tissue damage seen in cases of AMI known to be secondary to a thrombotic process taking place in the mesenteric veins [12]. In contrast, arterial AMI has a pathologic pattern where areas of dark, black affected tissue are sharply demarcated from unaffected tissue [12]. Furthermore, the presence of similar lesions in multiple areas supplied by different vasculature further supports the idea that the patient experienced an episode of widespread coagulopathy.

Immunostaining techniques were employed in this case to further evaluate the discovered malignancy and revealed the tumor stained negative for TTF-1 and positively stained for CD56, chromogranin A, synaptophysin, pan keratin (AE1/AE3), and CDX2; this pattern of immunohistochemistry staining indicated that this is likely a primary neuroendocrine tumor of the GI tract, most likely originating from the foregut or midgut [13]. As stated before, cancer patients are at much higher risk than the general population of suffering a VTE event, with some studies stating that this increased VTE risk among cancer patients is around 4-7 times higher than the relative VTE risk of the general population [14]. Further compounding on this is additional research that found patients with metastatic cancers were 4-13 times more likely to experience a thrombotic event than those with localized cancers [14]. Certain cancers like mucin-secreting gastric and pancreatic adenocarcinomas are believed to place patients at the highest increased risk for VTE, but recent studies have found that GI neuroendocrine tumors also have been associated with an increased VTE risk, albeit not as severe as seen with their adenocarcinoma counterparts [14].

Based on that information, it is likely that this patient’s malignancy led to a hypercoagulable state, and it is possible that this coagulopathy was then superimposed upon by inflammation induced by the patient’s Salmonella enteritis. As discussed earlier, Salmonella LPS via the Shwarzman reaction can result in the sensitization of tissue to future cytokine stimulation and can also prompt “leukocytic consolidations around blood vessels, intravascular thrombosis, and the necrosis of venules [8].” Furthermore, it is believed that intestinal infarction resulting from MVT requires thrombotic involvement at the level of small perforating venules that then swell from venous outflow obstruction, leading to an increase in pressure that causes occlusion of corresponding arterioles [15]. It is possible that the aberrant clotting and thrombosis that is believed to have occurred in this patient may have been more pronounced in the patient’s GI tract due to the sensitization that resulted from the repeated exposures to Salmonella LPS during the patient’s retracted bout of enteritis; however, more research on this topic is needed for a definitive conclusion to be drawn.

This case demonstrates the complexity imposed by AMI and how its sometimes-insidious onset and nonspecific symptoms can coalesce with patient risk factors and comorbidities, often leading to diagnostic delay and more advanced ischemia. Clinicians in the future could work to decrease diagnostic delays incases of AMI, especially in cases due to MVT, by maintaining a high degree of clinical suspicion for this condition, considering ruling out thrombotic disorders in other organ systems as well while evaluating a patient for PE or DVT in the setting of possible coagulopathy, and by potentially more readily utilizing alternative advanced imaging modalities if traditional CT or computed tomographic angiography (CTA) are contraindicated or unavailable. In this case, management of this patient was complicated by the patient’s intolerance to iodinated CT contrast. The diagnoses of AMI and MVT both heavily rely on contrast-aided imaging; recent improvements in CT technology and availability have led to biphasic contrast-enhanced CT with three-dimensional multiplanar reconstruction being the preferred imaging modality for evaluating possible cases of AMI due to its high sensitivity and specificity of 93% and 100%, respectively [2]. Importantly, imaging for AMI should include both the arterial and venous phases; without a specific venous imaging phase, evidence of AMI due to MVT may be missed [2]. In cases of possible AMI occurring in patients for whom CTA is contraindicated or unavailable, clinicians can instead utilize gadolinium contrast-enhanced magnetic resonance angiography or magnetic resonance venography (MRA/MRV) to evaluate for the presence of arterial or venous occlusion [16].

Alternatively, in cases where both CTA and MRA/MRV are unavailable or not feasible, Doppler ultrasound (US) may be employed with excellent specificity (100%) but with variably less sensitivity (70%-90%) than CT or MRA/MRV [17]. However, Doppler US is highly dependent on operator proficiency and is not as successful at viewing MVT localized to smaller mesenteric veins and venules, so earlier cases of AMI due to MVT may be missed in those situations, potentially leading to a diagnostic delay [17].

Laboratory findings, like an increased serum lactate or D-dimer level, in cases of possible AMI can be helpful in increasing clinical suspicion for the condition or provide supporting information that points toward an increased likelihood that a patient presenting with abdominal pain is experiencing their symptoms as a result of AMI; however, lab markers are nonspecific and are overall nondiagnostic on their own [1]. Very marked elevations in serum lactic acid may be seen in cases of AMI with advanced ischemia and leukocytosis may be seen as well; the presence of a leukocytosis as seen here frequently portends a worse prognosis and often is accompanied by peritonitis and intestinal perforation [1,2]. Furthermore, in cases of AMI due to MVT, evidence of serum markers suggesting a hypercoagulable state (i.e., elevated D-dimer) may be present, but these show little efficacy for use as isolated screening tools when evaluating for intestinal ischemia due to their lack of specificity; it is possible they function best in this context at increasing clinical suspicion for a venous thrombotic process and support a decision to procure more advanced imaging like CTA or MRA/MRV [18].

Treatment of AMI is multifaceted, and the primary initial goal of treatment is restoration of normal intestinal perfusion. Fluid resuscitation is a mainstay of treatment and surgical intervention is almost always required to assess the degree of tissue damage and revascularize or resect affected areas [18]. However, in some cases of mild to moderate MVT that has not yet progressed to infarction and or peritonitis, less invasive treatments may be preferred; in these cases, treatment with IV anticoagulants like heparin or with localized thrombolytic infusions by interventional radiology (IR) has been associated with better patient outcomes than those seen in patients who underwent more traditionally invasive surgical treatments [18]. Once a patient suffering from MVT has shown stable clinical improvement, a transition from heparin to warfarin therapy can be initiated and should be continued for at least six months in cases where the cause of the thrombosis is known and reversible; patients with an unknown cause for their hypercoagulable states should stay on systemic anticoagulation therapy for the remainder of their lifetime [18]. In the case of this patient, surgical site infections (SSIs) are very likely, as there is an increase in their incidence in patients undergoing colorectal surgery [19]. Risk factors include diabetes, steroid usage, BMI over 30, and American Society of Anesthesiologists (ASA) class greater than two [19]. Due to initiation of standard sepsis protocol, steroids were given and likely contributed to the numerous postsurgical infections she had as well as her ASA classification. Therefore, providers must be vigilant of SSIs in this population, as they lead to increased morbidity, mortality, and length of hospital stay [19].

## Conclusions

In conclusion, AMI is a severe and potentially lethal medical condition that is occurring with increased frequency in the United States. This case report illustrates how AMI, especially cases caused by MVT, can present insidiously and with initially nonspecific symptoms before rapidly progressing into a patient’s abrupt clinical decompensation. The information provided here demonstrates that clinicians evaluating patients with abdominal pain should consider AMI as a part of their differential diagnosis, especially in patients who are 70 years of age or older, reporting pain out of proportion with physical exam findings, and/or those with a history of atherosclerotic or hypercoagulable risk factors. If CT with contrast is not available, MRA and MRV are acceptable alternatives and should be used in patients with high clinical suspicion for mesenteric venous ischemia.
